# Whole-Genome Comparison Reveals Heterogeneous Divergence and Mutation Hotspots in Chloroplast Genome of *Eucommia ulmoides* Oliver

**DOI:** 10.3390/ijms19041037

**Published:** 2018-03-30

**Authors:** Wencai Wang, Siyun Chen, Xianzhi Zhang

**Affiliations:** 1 Institute of Clinical Pharmacology, Guangzhou University of Chinese Medicine, Guangzhou 510000, China; wencaiwang@gzucm.edu.cn; 2 Germplasm Bank of Wild Species, Kunming Institute of Botany, Chinese Academy of Sciences, Kunming 650201, China; chensiyun@mail.kib.ac.cn; 3 College of Forestry, Northwest A&F University, Yangling 712100, China

**Keywords:** *Eucommia ulmoides*, chloroplast genome, heterogeneous divergence, mutation hotspots, whole-genome comparison

## Abstract

*Eucommia ulmoides* (*E. ulmoides*), the sole species of Eucommiaceae with high importance of medicinal and industrial values, is a Tertiary relic plant that is endemic to China. However, the population genetics study of *E. ulmoides* lags far behind largely due to the scarcity of genomic data. In this study, one complete chloroplast (cp) genome of *E. ulmoides* was generated via the genome skimming approach and compared to another available *E. ulmoides* cp genome comprehensively at the genome scale. We found that the structure of the cp genome in *E. ulmoides* was highly consistent with genome size variation which might result from DNA repeat variations in the two *E. ulmoides* cp genomes. Heterogeneous sequence divergence patterns were revealed in different regions of the *E. ulmoides* cp genomes, with most (59 out of 75) of the detected SNPs (single nucleotide polymorphisms) located in the gene regions, whereas most (50 out of 80) of the indels (insertions/deletions) were distributed in the intergenic spacers. In addition, we also found that all the 40 putative coding-region-located SNPs were synonymous mutations. A total of 71 polymorphic cpDNA fragments were further identified, among which 20 loci were selected as potential molecular markers for subsequent population genetics studies of *E. ulmoides*. Moreover, eight polymorphic cpSSR loci were also developed. The sister relationship between *E. ulmoides* and *Aucuba japonica* in Garryales was also confirmed based on the cp phylogenomic analyses. Overall, this study will shed new light on the conservation genomics of this endangered plant in the future.

## 1. Introduction

There are profuse paleoendemics (e.g., Eucommiaceae) and/or phylogenetically primitive taxa (e.g., Cercidiphyllaceae) in China due to the glaciation refuge role played during the Quaternary period [1,2]. Unfortunately, up to *circa* 5000 flora species are currently endangered in China, some of which have already become extinct [3]. Many plant species with important medicinal values have also been threatened seriously due to the increasing demand for raw materials of medicines, over-harvesting and habitat-loss [4,5,6]. Conservation of medicinal plants has become one of the most urgent issues faced today in China.

*Eucommia ulmoides* Oliver, a dioecious woody plant endemic to China, is the sole species in the family Eucommiaceae [7]. *E. ulmoides* has been widely cultivated and used as a herbal drug to reduce blood pressure and strengthen the body in central and southern China for at least 2000 years [8,9]. *E. ulmoides* is also well-known as a “hardy rubber” tree that produces trans-polyisoprene rubber (i.e., gutta or Eu-rubber) in the leaves, bark, and pericarp [10,11]. It has been shown that *Eucommia* fossils occurred widely across the Northern Hemisphere from the Palaeocene onwards [12], which indicates that *E. ulmoides* is a representative model of Tertiary relict species, i.e., living from Tertiary to present. However, *E. ulmoides* may have been extinct in the wild and already listed in the Red List of Endangered Plant Species in China probably due to exhaustive human exploration [13,14]. Therefore, effective strategies are urgently needed to conserve this rare and endangered medicinal plant.

To date, studies on *E. ulmoides* have mainly focused on the morphological variation and the natural products [8,15]. Molecular and population genetics studies of this valuable tree lag behind largely due to limited DNA sequence resources [16,17]. Recently, nuclear microsatellites (nrSSR) were developed to investigate the genetic diversity of *E. ulmoides* [18,19]. Amplified fragment length polymorphism (AFLP) and sequence-related amplified polymorphism (SRAP) have been used to construct genetic maps of *E. ulmoides* [20,21]. The genetic markers of random amplified polymorphic DNA (RAPD), chloroplast microsatellite (cpSSR) and inter-simple sequence repeat (ISSR) have also been uncovered [22,23,24]. Nevertheless, the variability of these developed fingerprinting markers in *E. ulmoides* is relatively low, with limited population genetics information. A new and promising marker type i.e., Single Nucleotide Polymorphism (SNP) has gained high popularity during the last two decades [25,26]. With the on-going progress of high throughput sequencing techniques, it has become convenient to collect large-scale SNP data for genetic analyses [27,28]. Using SNP markers in conservation genetics studies of endangered plants has attracted much attention; for instance, in *Pinus ponderosa* Douglas ex Lawson [29], and *Sciadopitys verticillata* (Thunb.) Siebold and Zucc [30].

Chloroplast (cp) DNA sequences have been extensively used in the studies of plant population genetics and molecular phylogenetics [31,32,33]. Typically, cp genomes of land plants have a quadripartite structure with a pair of inverted repeats (IRs) separating a large single-copy (LSC) region and a small single-copy (SSC) region, ranging from 115 to 165 kilobase (kb) [34]. The cp genomes in general are inherited uniparentally, mostly maternally and are essentially recombination-free, leading to a smaller effective population size and a shorter coalescent time than the nuclear genomes [35]. Recently Wang et al. [17] reported a cp genome sequence of *E. ulmoides* with a length of 163,341 bp. Clearly, the availability of additional sequenced cp genomes from *E. ulmoides* would aid our understanding of the cp genome-wide variation at the individual level. Through comparative genomic analysis, polymorphic cpDNA loci with plentiful SNPs and indels i.e., nucleotide insertions and deletions can also be detected, which would be useful for further population genetics studies of *E. ulmoides*.

Genome skimming is currently one of the most economical techniques to obtain plastome sequences [36], through which obtaining complete cp genomes for plant phylogenomics inference becomes convenient [37]. In this study, we generated and characterized one complete cp genome of *E. ulmoides* using the genome skimming approach. By comparing the cp genome generated in this study and the one published previously [17], our main goals were to: (1) test whether the cp genomes in *E. ulmoides* show structural rearrangements; (2) reveal the divergence pattern of the cp genome in *E. ulmoides*; (3) identify highly variable cp genome-wide markers for subsequent population genetics studies of *E. ulmoides*.

## 2. Results

### 2.1. Chloroplast Genome Variation in E. ulmoides

About 20 million clean reads (4.72 Gb data) were generated from genome skimming sequencing. Two assembly methods (CLC Genomics Workbench and SPAdes software) both obtained the complete cp genome of *E. ulmoides* with high genome coverage (>180×) and there is no difference between the two assembled sequences, suggesting a high-quality cp genome map was achieved. The final cp genome size was determined to be 163,586 bp, similar to the previously published one (KU204775) (Table 1). The number of protein-coding genes, tRNA genes and rRNA genes, were the same as those in the available *E. ulmoides* cp genome (KU204775). We have deposited the newly sequenced *E. ulmoides* cp genome in GenBank with accession number MF766010. The genome skimming sequencing reads have also been deposited in the Sequence Read Archive (SRA) with the accession number PRJNA399774.

**Figure 1:**
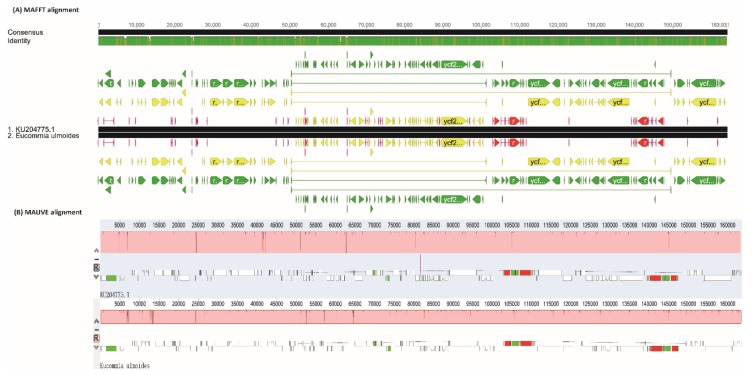
Conserved chloroplast genome structure in *Eucommia ulmoides*. (**A**) Pairwise chloroplast genome alignments derived from Multiple Alignment using Fast Fourier Transform (MAFFT) program. The sequence identity is indicated on the top. Label KU204775.1 represents the *E. ulmoides* chloroplast genome retrieved from GenBank, while label *E. ulmoides* indicates the newly sequenced genome in this study. (**B**) Pairwise chloroplast genome alignments derived from MAUVE software.

The whole genome alignments from MAFFT (Figure 1A) and MAUVE (Figure 1B) were consistent. There were no large genome rearrangements in the two cp genomes, which indicated that the cp genome structure in *E. ulmoides* is highly conserved and perfectly syntenic (Figure 1). Interestingly, small-scale nucleotide insertions and deletions were detected in the *E. ulmoides* cp genome. We found 15 insertions with more than ten nucleotides (11–111 bp) in the two cp genomes (Table 2). Five deletions in the range of 16–90 bp were also uncovered. It is worth noting that all of these insertions and deletions were involved in repeat sequence expansions and contractions (Table 2). Furthermore, across the entire cp genome of *E. ulmoides*, the sequence divergences were not uniform but highly heterogeneous (Figure 1).

### 2.2. Molecular Marker Development

**Figure 2:**
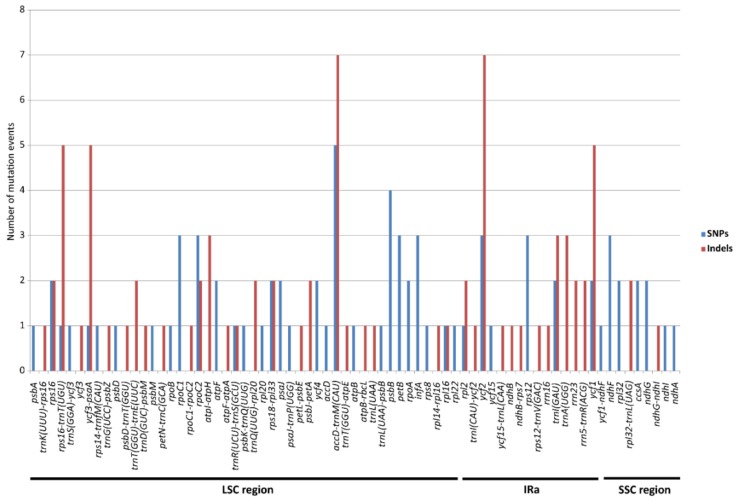
Mutational events (SNPs and indels) detected across the chloroplast genome of *Eucommia ulmoides*. SNPs (single nucleotide polymorphisms) indicate nucleotide substitutions and indels represent nucleotide insertions and deletions. The homologous loci are oriented according to their locations in the chloroplast genome.

A total of 155 mutational events, including 75 nucleotide substitutions (SNPs) and 80 nucleotide indels (insertions and deletions), were detected within 71 loci of the cp genome in *E. ulmoides* (Figure 2). There were 98 mutations (51 SNPs and 47 indels) and 15 mutations (12 SNPs and 3 indels) in the LSC and SSC regions, respectively. In addition, 42 mutations (12 SNPs and 30 indels) were located in the IR region. Distribution patterns of SNPs and indels differed largely in the cp genic and intergenic regions of *E. ulmoides*. Most of the SNPs (59 out of 75) were found in the gene sequences, including 31 protein-coding genes and one tRNA gene. In contrast, indels were mainly (50 out of 80) distributed in the intergenic spacers (Figure 2). Upon further investigation of SNPs and indels in the nine intron-containing protein-coding genes (*atpF*, *ndhA*, *ndhB*, *rpl2*, *rpl16*, *rpoC1*, *rps12*, *rps16*, *ycf3*), we found that all the mutations were located in the intron regions. In all, 40 SNPs and 14 indels occurred in the plastid-coding sequence (CDS) regions.

**Figure 3:**
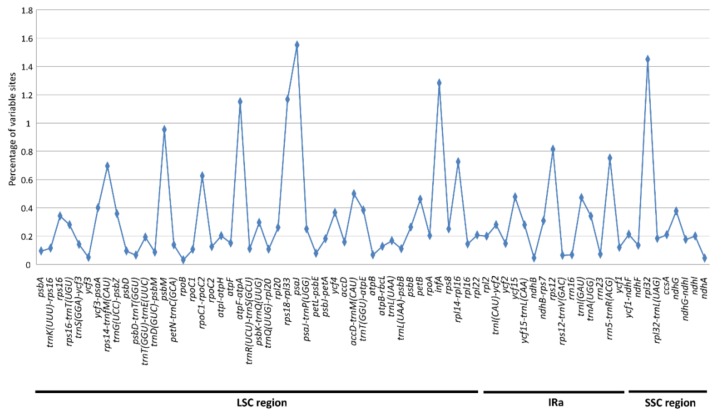
Percentage of variable characters (SNPs and indels) in polymorphic chloroplast loci in *Eucommia ulmoides*. The homologous loci are oriented according to their locations in the chloroplast genome.

The proportion of variability in the 71 polymorphic loci ranged from 0.03% to 1.55% with a mean value of 0.37% (Figure 3). The mutation rates in most (53 out of 71) of the loci were between 0.10 and 1.00%. Five of these DNA fragments i.e., *atpF-atpA*, *rps18-rpl33*, *psaJ*, *infA* and *rpl32* had variations exceeding 1.00%. Considering the relatively high percentage of variability and convenience for primer design in PCR (Polymerase Chain Reaction) and sequencing experiments, we chose 20 highly variable loci with length of 200–1500 bp as potential molecular markers for subsequent population genetic studies (Table 3). The percentage of variations in these 20 loci all exceeded 0.25%, among which 16 had a percentage of variable characters (VCs) greater than 0.30% (Table 3).

Through SSR analysis, we found a total of 31 SSR loci in the newly assembled *E. ulmoides* cp genome, among which 27 were shared by the two genomes. Further detection revealed that eight cpSSR loci were polymorphic in *E. ulmoides* (Table 4). All the polymorphic cpSSR loci were mononucleotide repeats, ranging from 10–15 bp in length. Five polymorphic cpSSR loci were located in the LSC region, with another three ones in the IR regions (Table 4).

### 2.3. SNP Calling and Phylogenomic Inference

The SNPs calling analysis using the previously published *E. ulmoides* cp genome (KU204775) as reference revealed a total of 75 SNPs. This result of SNP occurrence was consistent with the aforementioned molecular marker analysis (75 SNPs, Figure 2), which indicated that the detected SNPs were really present in different individuals of *E. ulmoides*. Further examination of the 40 SNPs in the CDS regions suggested that all these SNPs were synonymous, i.e., no amino acid change at the protein level. There were 34 transitions and six transversions in the protein-coding region SNPs. The average frequency of SNPs occurrence in the *E. ulmoides* cp genome was calculated as 0.46 per kb.

**Figure 4:**
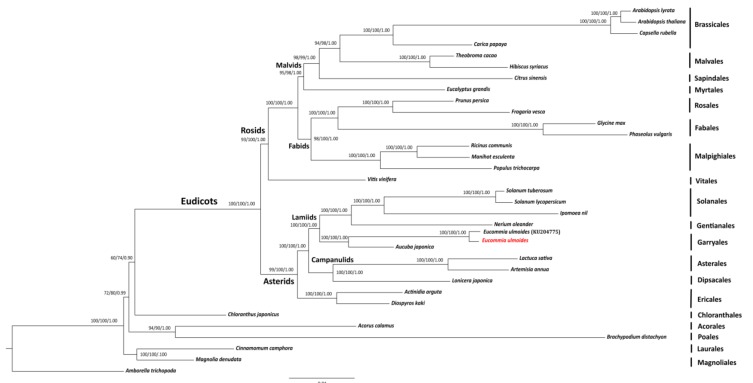
Maximum likelihood (ML) tree for 34 taxa based on 80 unique plastid protein-coding genes of *Eucommia ulmoides*. Values above the branches represent maximum parsimony bootstrap (MPBS)/maximum likelihood bootstrap (MLBS)/Bayesian inference posterior probability (PP). The newly sequenced *Eucommia ulmoides* chloroplast genome is indicated by red color and the previously published *E. ulmoides* chloroplast genome is followed by its GenBank accession number KU204775.

The final length of the supermatrix dataset contained 96,894 unambiguously aligned nucleotide characters. Three methods produced a congruent phylogenetic tree, shown in Figure 4. Eudicots, monocots and magnoliids were all highly supported to be monophyletic (94–100/90–100/1.00). Magnoliids diverged firstly, followed by monocots, then Chloranthales and eudicots, with relatively low (60–72/74–80/0.90–0.99) support values. Two *E. ulmoides* individuals clustered together with high statistical support values (100/100/1.00), which was subsequently sister to *Aucuba japonica* Thunb. in the Garryales clade with high statistical support values (100/100/1.00). Garryales, Gentianales and Solanales formed as the highly supported lamiids lineage (100/100/1.00), resolved as (Garryales, (Gentianales, Solanales)). Asterales and Dipsacales formed a highly supported clade, campanulids (100/100/1.00), which was sister to lamiids (100/100/1.00) in asterids. Ericales was resolved as the basal most group in the asterids lineage (100/100/1.00). Brassicales, Malvales, Myrtales and Sapindales clustered in the malvids clade (95/98/1.00) with resolution as (((Brassicales, Malvales), Sapindales), Myrtales). Fabales, Malpighiales and Rosales were located in the fabids lineage (98/100/1.00), which was a sister clade to malvids in rosdis.

## 3. Discussion

### 3.1. Conserved Chloroplast Genome Structure in E. ulmoides

Land plant cp genomes are generally inherited as a haplotype with no recombination, providing useful genetic information to trace relationships between different species [35,38]. Within species cp genome structure was highly conserved [39]. As expected, it is the case in *E. ulmoides* in terms of the contained genes and coding regions in the cp genomes (Table 1). Further whole-genome alignments suggested that the two cp genomes of *E. ulmoides* did not show genome rearrangement having the same linear gene order (Figure 1). As such it is reasonable to use cp genomes for subsequent conservation genomics studies on *E. ulmoides*.

It is noteworthy that the newly sequenced cp genome of *E. ulmoides* (163,586 bp) in this study is 245 bp larger than that of the previously reported one (163,341 bp, [17]) (Table 1). The cp genome size variation within different individuals of the same species has been reported for several other plants, such as in *Camptotheca acuminate* Decne. with its size varied as 157,806 bp [40], 157,877 bp [41] and 162,382 bp [42]. Nuclear genome size variations in plants are mostly caused by the repeats activities (e.g., expansions/contractions) via illegitimate recombination in addition to polyploidy [43,44,45,46,47]. In the two *E. ulmoides* cp genomes, we detected 15 insertions and five deletions, with more than 10 nucleotides for each (Table 2). All these sequences were observed to be part of or the whole DNA repeats. For instance, the repeat sequences in *rps16-trnT(UGU)*, *psbD-trnT(GGU)*, *rps12-rpl20* and *accD-trnM(CAU)* have been detected in the study of Wang et al. [17] as well. Therefore, potentially the illegitimate recombination between repeat regions of *E. ulmoides* cp genome may contribute to the cp genome size variation.

### 3.2. Heterogeneous Divergence in E. ulmoides Chloroplast Genome

Heterogenous divergence patterns in cp genomes have been reported in several plant groups, such as in Actinidiaceae [37] and in Poaceae [48]. The alignment of the two available cp genomes of *E. ulmoides* revealed highly heterogeneous sequence divergences within this species (Figure 1 and Figure 2). All the identified SNPs and indels from the intron-containing protein-coding genes were located in the intron regions. Due to natural selection CDS regions are in general more conserved than non-coding regions (i.e., intergenic sequences and introns) [49]. In addition, nucleotide substitutions likely have less destructive effect to the integrity of open reading frame (ORF) than indels [50]. We, thus, speculated that this functional constraint may lead to the contrasting occurrences of SNPs and indels in the *E. ulmoides* cp genomes.

The occurrence of synonymous SNPs was more abundant than that of the non-synonymous SNPs in CDS regions because of selection process [51,52]. As expected, all the SNPs detected in protein-coding regions were synonymous. Moreover, since the transitions rather than the transversions usually would generate more synonymous mutations in the CDS the transitions SNPs are more easily retained than the transversion ones [52,53]. In this study we found that the transition SNPs (34) were indeed more frequently detected than the transversion SNPs (6). Previous studies have reported a high level of nuclear genetic diversity at the population level of *E. ulmoides* [18,54]. In this study, we revealed that the frequency of plastid SNPs were 0.46 per kb at the whole cp genome level, lower than the average of 1.02 per kb in the nuclear genes of *E. ulmoides* [54]. The difference of SNP frequency between the cp genome and the nuclear genes could be caused by insufficient sampling in this study and/or different variation rates between the plastid and nuclear sequences.

### 3.3. Mutation Hotspots in E. ulmoides Chloroplast Genome

In general, protein-coding genes in the cp genome have lower sequence variation than the non-coding loci, for instance in bamboos [55] and mimosoid legume [56]. However, an accelerated variation rate of some plastid protein-coding genes has been reported, such as the *psb* in Poaceae [51], *rps* in Saxifragales [57], and *accD* and *rpl20* in Actinidiaceae [37]. In *E. ulmoides*, we found three protein-coding genes (*infA, psaJ* and *rpl32*) that varied the most quickly, all having variations exceeding 1.2% (Figure 3). The gene *infA* encodes translation initiation factor 1 and has been found missing in cp genomes of several plant lineages, e.g., in rosids [58]. The other two genes i.e., *psaJ* and *rpl32* code for photosystem I protein J and ribosomal protein L32, respectively, both of which are short in length with the former having 129 bp and the latter 138 bp (this study and [17]). The relatively high level of variation of these genes in *E. ulmoides* indicates that they are less constrained. Abnormal DNA replication, repair or recombination [59,60] may lead to the elevated divergence of these genes. 

The genetic markers of SSR, AFLP and SRAP have been developed and used for the population genetics studies of *E. ulmoides* [18,20,21]. However, the above fingerprinting markers [23,24] may provide insufficient genetic information to resolve the population structure and history of *E. ulmoides*. The relationships among natural and cultivated populations of *E. ulmoides* are elusive at present [18,24]. SNP markers are ample in plant cp genomes, making them useful candidates for population genetics studies. Using cp genome SNP markers has widely received attention during the past few years with the advances of high throughput sequencing techniques [61,62]. Highly variable cpDNA fragments have been mined for phylogenetic and population genetic studies in several species using cp genomes data, such as in kiwifruit [37] and temperate woody bamboos [55]. Given that it would be easy to amplify and sequence DNA fragments with length from *circa* 200 to 1500 bp using Sanger sequencing method [62,63], we thus chose 20 cpDNA loci with relatively high genetic divergences as potential molecular markers (Table 3) for subsequent population genomics studies of *E. ulmoides*. These selected plastid genome-wide loci would genetically be informative for uncovering the genetic relationships among the natural and cultivated *E. ulmoides* populations.

Additionally, 27 cpSSR loci identified by Wang et al. [17] were also confirmed in our SSR analysis, among which eight were further mined as polymorphic cpSSR loci (Table 4). Given that polymorphic cpSSR loci could be applied as useful markers to meet certain study purposes under the circumstances of limited budget [64,65], the newly developed polymorphic cpSSR loci in *E. ulmoides* here would be potential genetic markers to facilitate subsequent population genetics studies in the future.

### 3.4. Phylogenomic Validation of E. ulmoides

The newly obtained *E. ulmoides* cp genome was further validated via phylogenomic analyses using 34 complete plastomes from 10 major lineages of angiosperms. The resulting phylogenomic tree highly supported the clade of two *E. ulmoides* cp genomes (Figure 4), confirming the validity of the assembled and annotated cp genome of *E. ulmoides* in this study. The sister relationship between *E. ulmoides* and *A. japonica* in the Garryales clade was highly supported, which is consistent with the results derived from five organellar genes [66] and 36 plastid genes [17], supporting the classification of *E. ulmoides* (Eucommiaceae) in the updated APG IV system [67]. *E. ulmoides* and *A. japonica* are both woody and have unisexual flowers in separate individuals, which seem to be morphological synapomorphies for the order Garryales [68]. An average of 92.6% identities between 78 common unique cp protein-coding genes in *E. ulmoides* and *A. japonica* were also detected, suggesting a high similarity between the two species at the molecular level. Garryales was shown to be closely related to the clade of (Gentianales + Solanales) in lamiids, in line with the APG IV system [67].

All 20 sampled orders were highly supported to be monophyletic separately (Figure 4), agreeing with the APG IV system [67]. Within eudicots two large sister clades i.e., asterids and rosids were uncovered, and the relationships among these two lineages were highly resolved as ((campanulids, lamiids), Ericales) and ((fabids, malvids), Vitales), respectively as stated previously [69]. The branching patterns of species within campanulids and lamiids are consistent with recent studies [66,70] as (((Gentianales, Solanales), Garryales), (Asterales, Dipsacales)). Our analyses also resolved the phylogenetic relationships within fabids and malvids as ((((Brassicales, Malvales), Sapindales), Myrtales), ((Fabales, Rosales), Malpighiales)), consistent with the results of previous studies [17,69]. It is noteworthy that the branching orders of magnoliids, monocots, Chloranthales and eudicots only obtained low-level support values here (Figure 4). Further studies with expanded taxon samples are expected to confirm the phylogeny of these lineages. Moreover, plastome is inherited uniparentally in general, which might introduce biases to species phylogeny inference [71,72]. Analyses using orthologous nuclear genes are also needed for studying the evolutionary history of *E. ulmoides* among the flowering plants [54].

## 4. Materials and Methods

### 4.1. Plant Materials and DNA Sequencing

Fresh healthy leaves were collected from an adult male individual of *E. ulmoides* growing in the Arboretum of Northwest Agricuture and Forest University in Yangling, Shanxi, China, in April 2015. After collection, the leaves were immediately immersed in liquid nitrogen and then stored at −80 °C until use. The voucher specimen of this tree was deposited at the Trees Herbarium of Northwest A and F University with accession number ZXZ15027.

Total genomic DNA was extracted by the CTAB method [73]. Paired-end (PE) libraries with insert size *circa* 500 bp were constructed from fragmented genomic DNA based on standard Illumina protocols (Illumina Inc., San Diego, CA, USA). Prepared library was then sequenced for PE 100 bp read length on the Illumina HiSeq 2000 platform at the Beijing Genomics Institute (BGI) in Shenzhen, China.

### 4.2. Genome Assembly and Annotation

Fastq format PE reads were supplied with adaptor sequences removed. Poor quality reads with phred scores lower than 20 for more than 10% of their bases were also removed. Two independent methods were used to assemble the *E. ulmoides* cp genome. (1) The cp genome was de novo assembled using the CLC Genomics Workbench v7.5 software (CLC Bio, Aarhus, Denmark) based on the clean reads. After discarding contigs with length <300 bp and sequences with coverage <50, the remaining contigs were searched against the available cp genome of *E. ulmoides* (GenBank accession number KU204775) that used as the reference by BLAST (http://blast.ncbi.nlm.nih.gov/) with *e*-value <10^−5^. Aligned contigs with ≥90% similarity and query coverage were determined as cpDNA sequences and ordered according to the reference genome. Small gaps were filled using PE clean reads as conducted in Wang et al. [37]. (2) The clean reads were firstly mapped to the reference cp genome of *E. ulmoides* to determine the proportion of cpDNA using Bowtie v2.3.1 program [74] with a maximum of 3 mismatches. Subsequently, we applied SPAdes v3.9 software [75] with default setting to assemble the cp genome using the determined cpDNA clean reads.

DOGMA software [76] was used for initial cp genome annotation. Start/stop codons and intron/exon boundaries were checked and adjusted manually when necessary by comparing to the reference genome. tRNA genes were confirmed based on tRNAscan-SE 1.21 [77].

### 4.3. Genome-Wide Comparison and Divergent Hotspot Identification

The previously published cp genome of *E. ulmoides* (accession number: KU204775) was downloaded from GenBank database (https://www.ncbi.nlm.nih.gov/genbank/). This genome was aligned with the *E. ulmoides* cp genome described herein, using MAFFT program [78] and MAUVE software [79], respectively, and manually adjusted where necessary. The obtained pairwise alignment of the cp genomes was visualized in Geneious v9.0 [80]. Moreover, given the genome repeat sequences expansion and contraction may result in genome size variation [81], we examined the DNA insertions and deletions in repeat regions of the two *E. ulmoides* cp genomes.

The two *E. ulmoides* cp genomes were analyzed to identify molecular markers that can be selected in subsequent population genetic studies. We firstly extracted both the genic and intergenic DNA fragments in each cp genome using the “Extract Sequences” option in DOGMA [76]. Then the homologous loci were aligned individually by MUSCLE program (http://www.drive5.com/muscle/) [82] implemented in Geneious v9.0 [80] with default settings. Manual adjustments were made for the alignments where necessary. The proportion of mutational events for each genic and intergenic locus was calculated as follows: the proportion of variation = ((NS + ID)/L) × 100, where NS = the number of nucleotide substitutions (SNPs), ID = the number of indels (insertions and deletions), L = the aligned sequence length.

Polymorphic cpSSR loci were further mined by genome comparison. Firstly, SSRs in the newly sequenced *E. ulmoides* cp genome were detected by MISA perl script (http://pgrc.ipk-gatersleben.de/misa/). The parameter of minimum repeat unit was set as 10 for mono-, 6 for di-, and 5 for tri-, tetra-, penta-, and hexanucleotide SSRs [83]. Then, all the identified SSR loci were compared to the 29 cpSSR loci of Wang et al. [17] to develop polymorphic cpSSR markers in *E. ulmoides*.

### 4.4. SNPs Validation and Phylogenomic Analyses

To confirm the SNPs identified by the aforementioned cp genome alignment, we here mapped the genome skimming clean reads generated in this study to the previously published *E. ulmoides* cp genome (KU204775) [17] for SNP calling. Picard-tools v1.41 (http://broadinstitute.github.io/picard/) and samtools v0.1.18 [84] were applied to sort and remove duplicated reads and merge the bam alignment results. GATK3 software [85] was further used to perform SNPs identification. Raw vcf files were filtered with GATK standard filter method and other parameters were set as defaults. Moreover, to reveal if the coding region SNPs detected in *E. ulmoides* cp genome caused amino acid substitution on protein level, we firstly translated each protein-coding gene into amino acids in Geneious v9.0 [80]. Then the protein sequences of each gene were aligned, respectively using MUSCLE [82]. The mutational events were checked to uncover the synonymous and nonsynonymous SNPs and the nucleotide transitions and transversions.

Phylogenomic analyses were also conducted to validate the newly assembled and annotated *E. ulmoides* cp genome. 34 plastomes representing 10 major lineages of angiosperms (Table S1) were included for phylogenomic analyses. *Amborella trichopoda* from basal angiosperm lineages was defined as outgroup according to previous studies [66,67]. 80 unique plastid protein-coding genes of *E. ulmoides* (Table S2) were used for the phylogenetic inferences. Each gene was aligned individually by MUSCLE [82] in Geneious v9.0 [80], and then concatenated as a supermatrix. Gaps were not included in the dataset.

Three methods i.e., maximum parsimony (MP), maximum likelihood (ML), and Bayesian inference (BI) were used for phylogenetic reconstruction. We performed parsimony heuristic tree searches in PAUP v4.0b10 [86] with parameters set as 1000 random addition sequence replicates, tree bisection and reconnection (TBR) branch swapping, and MulTrees option in effect. 1000 bootstrap replicates [87] were calculated to evaluate the branch support (MPBS) of the MP tree. RAxML v.8.2.8 [88] and MrBayes 3.2.6 [89] in the CIPRES Science Gateway v3.3.3 [90] were applied for ML and BI analyses, respectively. Supermatrix was partitioned by genes and GTR + G model of nucleotide substitution was used. For the ML tree we conducted 1000 fast bootstrap ML reps to assess the support values (MLBS) of internal nodes. In Bayesian analysis two runs with four chains were carried out up to 50,000,000 generations, sampling one tree every 1000 generations till convergence, i.e., the average standard deviation of split frequencies <0.01. We discarded the first 25% of trees as burn-in, and used the remaining trees to estimate the majority-rule consensus BI tree and posterior probabilities (PP).

## 5. Conclusions

In summary, in the present study we generated one complete cp genome of *E. ulmoides* using the genome skimming approach. Through comprehensive genome-wide comparative analyses we found that the cp genomes within *E. ulmoides* were highly conserved in terms of structure and content. Nevertheless, obviously heterogeneous sequence divergences were revealed in different regions of the *E. ulmoides* cp genome. A total of 20 polymorphic DNA fragments and eight SSR loci have been identified as potential cpDNA markers for subsequent population genetics studies of this tree species. The phylogenetic placement of *E. ulmoides* in angiosperms was robustly resolved as well based on the cp genomes data, strongly supporting the sister relationship between *E. ulmoides* and *A. japonica* in the asterids lineage. The data presented here will aid further conservation genomic studies and facilitate the development of plastid genetic engineering for *E. ulmoides*.

## Figures and Tables

**Table 1:** Comparison between the newly and previously sequenced chloroplast genomes of *Eucommia ulmoides*.

Item	This Study	KU204775
Chloroplast genome size (bp)	163,586	163,341
LSC ^a^ length (bp)	86,764	86,592
SSC ^b^ length (bp)	14,166	14,149
IRa/IRb ^c^ length (bp)	31,328	31,300
Number of genes (unique genes)	136 (115)	136 (115)
Number of protein-coding genes (unique genes)	89 (80)	89 (80)
Number of tRNA genes (unique genes)	39 (31)	39 (31)
Number of rRNA genes (unique genes)	8 (4)	8 (4)
GC ^d^ content (%)	38.33%	38.34%
Protein-coding regions (%)	51.91%	51.99%

^a^ LSC, large single-copy region; ^b^ SSC, small single-copy region; ^c^ IRa/IRb, two identical inverted repeat regions a/b; ^d^ GC, Guanine and Cytosine.

**Table 2:** DNA insertions and deletions with more than 10 nucleotides in the chloroplast genomes of *Eucommia ulmoides*.

No.	Size (bp)	Start Position	Location	Type
1	56	6851	*rps16-trnT(UGU)*	insertion
2	27	7006	*rps16-trnT(UGU)*	insertion
3	45	7196	*rps16-trnT(UGU)*	insertion
4	13	12,693	*ycf3-psaA*	insertion
5	23	12,912	*ycf3-psaA*	insertion
6	111	13,312	*ycf3-psaA*	insertion
7	12	13,471	*ycf3-psaA*	insertion
8	32	24,279	*psbD-trnT(GGU)*	insertion
9	12	26,615	*trnD(GUC)-psbM*	insertion
10	11	51,194	*rps12-rpl20*	insertion
11	17	52,547	*rps18-rpl33*	insertion
12	40	57,075	*psbJ-petA*	insertion
13	18	64,506	*accD-trnM(CAU)*	insertion
14	12	73,717	*trnL(UAA) intron*	insertion
15	14	127,486	*ndhG-ndhI*	insertion
16	16	4673	*trnK(UUU)-rps16*	deletion
17	44	24,700	*trnG(TTU)-trnE(UUC)*	deletion
18	31	41,767	*atpI-atpH*	deletion
19	44	51,109	*rps12-rpl20*	deletion
20	90	62,865	*accD-trnM(CAU)*	deletion

**Table 3:** The 20 chloroplast DNA fragments with relative high genetic divergences identified in *Eucommia ulmoides*.

Region	Aligned Length (bp)	No. VCs ^a^	Percentage of VCs (%)
*infA*	234	3	1.28
*rps18-rpl33*	343	4	1.17
*rps12*	369	3	0.81
*rrn5-trnR(ACG)*	266	2	0.75
*ycf15*	210	1	0.48
*trnI(GAU)*	1062	5	0.47
*petB*	651	3	0.46
*ycf3-psaA*	1502	6	0.40
*trnT(GGU)-atpE*	261	1	0.38
*ndhG*	531	2	0.38
*ycf4*	546	2	0.37
*trnG(UCC)-psbZ*	280	1	0.36
*rps16*	1170	4	0.34
*trnA(UGG)*	881	3	0.34
*ndhB-rps7*	325	1	0.31
*psbK-trnQ(UUG)*	338	1	0.30
*trnI(CAU)-ycf2*	357	1	0.28
*ycf15-trnL(CAA)*	359	1	0.28
*psbB*	1521	4	0.27
*rpl20*	384	1	0.26

^a^ VCs: variable characters, including SNPs and indels.

**Table 4:** The polymorphic chloroplast SSRs identified in *Eucommia ulmoides*.

No.	SSR Repeat Motif	Length Variation (bp)	Location	Region ^a^
1	(G)	10–11	*trnG(UCC)-psbZ*	LSC
2	(A)	12–15	*rpoC2*	LSC
3	(A)	12–13	*ycf1*	IRb
4	(A)	13–14	*rpl32-trnL(UAG)*	IRa
5	(T)	10–14	*psbJ-petA*	LSC
6	(T)	10–11	*trnG(GCC)-trnS(GCU)*	LSC
7	(T)	12–13	*ycf1*	IRa
8	(T)	14–15	*rpl16-rps3*	LSC

^a^ LSC, large single-copy region; IRa/IRb, two identical inverted repeat regions a/b.

## Supplementary Materials

The following are available online at http://www.mdpi.com/1422-0067/19/4/1037/s1. Table S1: Taxa and GenBank accession numbers included in the phylogenomic analyses, Table S2: List of 80 unique plastid protein-coding genes of *Eucommia ulmoides* included in the phylogenomic analyses.
