# Supplementary material for: Whole-Genome Comparison Reveals Heterogeneous Divergence and Mutation Hotspots in Chloroplast Genome of Eucommia ulmoides Oliver
Source: Int J Mol Sci. 2018 Mar 30;19(4):1037. doi: 10.3390/ijms19041037 (PMC5979487; doi:10.3390/ijms19041037)
Supplement: Supplementary file 1 [file ijms-19-01037-s001.pdf]

Table S1. Taxa and GenBank accession numbers included in the phylogenomic analyses.

Classification	Taxon	GenBank Accession No.
Acorales/Acoraceae	*Acorus calamus*	NC_007407
Amborellales/Amborellaceae	*Amborella trichopoda*	NC_005086
Asterales/Compositae	*Artemisia annua*	NC_034683
	Lactuca sativa	NC_007578
Brassicales/Brassicaceae	*Arabidopsis lyrata*	NC_031186
	Arabidopsis thaliana	NC_030789
	Capsella rubella	NC_026839
Brassicales/Caricaceae	*Carica papaya*	NC_010323
Chloranthales/Chloranthaceae	*Chloranthus japonicus*	NC_026565
Dipsacales/Caprifoliaceae	*Lonicera japonica*	NC_026839
Ericales/Actinidiaceae	*Actinidia arguta*	NC_031186
Ericales/Ebenaceae	*Diospyros kaki*	NC_030789
Fabales/Fabaceae	*Glycine max*	NC_007942
	Phaseolus vulgaris	NC_009259
Garryales/Eucommiaceae	*Eucommia ulmoides* [a]	MF766010
	E. ulmoides	KU204775
Garryales/Garryaceae	*Aucuba japonica*	GQ997049-GQ997131
Gentianales/Apocynaceae	*Nerium oleander*	NC_025656
Laurales/Lauraceae	*Cinnamomum camphora*	NC_035882
Magnoliales/Magnoliaceae	*Magnolia denudata*	NC_018357
Malpighiales/Euphorbiaceae	*Manihot esculenta*	NC_010433
	Ricinus communis	NC_016736
Malpighiales/Salicaceae	*Populus trichocarpa*	NC_009143
Malvales/Malvaceae	*Hibiscus syriacus*	NC_026909
	Theobroma cacao	NC_014676
Myrtales/Myrtaceae	*Eucalyptus grandis*	NC_014570

Poales/Poaceae	*Brachypodium distachyon*	NC_011032
Rosales/Rosaceae	*Fragaria vesca*	NC_015206
	Prunus persica	NC_014697
Sapindales/Rutaceae	*Citrus sinensis*	NC_008334
Solanales/Convolvulaceae	*Ipomoea nil*	NC_031159
Solanales/Solanaceae	*Solanum lycopersicum*	NC_007898
	Solanum tuberosum	NC_008096
Vitales/Vitaceae	*Vitis vinifera*	NC_007957

[a] The newly sequenced *E. ulmoides* individual in the current study.

Table S2. List of 80 unique plastid protein-coding genes of *Eucommia ulmoides* included in the phylogenomic analyses.

accD, atpA, atpB, atpE, atpF, atpH, atpI, ccsA, cemA, infA, matK, ndhA, ndhB, ndhC, ndhD, ndhE, ndhF, ndhG, ndhH, ndhI, ndhJ, ndhK, petA, petB, petD, petG, petL, petN, psaA, psaB, psaC, psaI, psaJ, psbA, psbB, psbC, psbD, psbE, psbF, psbH, psbI, psbJ, psbK, psbL, psbM, psbN, psbT, psbZ, rbcL, rpl14, rpl16, rpl2, rpl20, rpl22, rpl23, rpl32, rpl33, rpl36, rpoA, rpoB, rpoC1, rpoC2, rps2, rps3, rps4, rps7, rps8, rps11, rps12, rps14, rps15, rps16, rps18, rps19, ycf1, ycf15, ycf2, ycf3, ycf4, ycf68
